# Magnetically-Actuated Mixing and Merging of Acid-Base Micro-Droplets on Open Surfaces: Preliminary Study

**DOI:** 10.3390/s18061767

**Published:** 2018-06-01

**Authors:** Mei-Kum Khaw, Faisal Mohd-Yasin, Nam-Trung Nguyen

**Affiliations:** 1 Queensland Micro-and Nanotechnology Centre, Griffith University, Nathan QLD 4111, Australia; khawmk@utar.edu.my (M.-K.K.); nam-trung.nguyen@griffith.edu.au (N.-T.N.); 2 Lee Kong Chian Faculty of Engineering and Science, Universiti Tunku Abdul Rahman, Bandar Sungai Long, Kajang 43000, Selangor, Malaysia

**Keywords:** microfluidics, micro-droplet, magnetic-actuation, micro-mixing, acid-base, exothermic reaction

## Abstract

We present the mixing and merging of two reactive droplets on top of an open surface. A mobile droplet (1.0 M HCl solution + iron oxide particles) is magnetically-actuated to merge with a sessile droplet (1.0 M NaOH + phenolphthalein). The heat from the exothermic reaction is detected by a thermocouple. We vary the droplet volume (1, 5 and 10 μL), the magnet speed (1.86, 2.79, 3.72 and 4.65 mm/s) and the iron oxide concentration (0.010, 0.020 and 0.040 g/mL) to study their influences on the mixing time, peak temperature and cooling time. The sampled recording of these processes are provided as supplementary files. We observe the following trends. First, the lower volume of droplet and higher speed of magnet lead to shorter mixing time. Second, the peak temperature increases and cooling time decreases at the increasing speed of magnet. Third, the peak temperature is similar for bigger droplets, and they take longer to cool down. Finally, we also discuss the limitations of this preliminary study and propose improvements. These observations could be used to improve the sensitivity of the open chamber system in measuring the exothermic reaction of biological samples.

## 1. Introduction

Micro-calorimeters are efficient tools to measure the heat of chemical reactions of small biological samples such as proteins, ligands and enzymes [1]. In many circumstances, these samples are not available or reproducible in a large amount [2]. To address this issue, researchers have proposed the use of a droplet-based instead of continuous flow-based microfluidic systems [1]. One of the major considerations is the mixing of reactive droplets [2]. Mixing can happen passively or actively. Passive mixing relies on geometric features and gravity, whereas active mixing relies on external forces [3,4]. Active mixing is favored due to the shorter mixing time [5]. The major mechanisms include electrostatically-actuated mixing, magnetically-actuated mixing and magnetic micro-stirring [1]. Electrostatically-actuated mixing depends on the combination of the electrostatic force and surface tension to merge the polarized droplets [6,7]. Magnetically-actuated mixing utilizes magnetic particles in one of the droplets. An external magnet being slid under the diamagnetic surface will move the droplet containing magnetic particles to merge with a sessile droplet. A computer program moves the magnet in repetitive forward and backward motions to mix them [8,9]. Magnetic micro-stirring is a combination of previous two mechanisms. The droplets are first merged electrostatically. The sessile droplet contains a stir bar instead of magnetic particles [10]. The mixing time is accelerated by a stir bar, which is controlled by a rotating magnet [11]. Alternatively, Roy et al. replaced the bar with rotating magnetic beads [12]. In some cases, the magnetically actuated mixing is preferred over the other two. Its mixing time is faster compares to electrostatic mixing, and it does not require an expensive fabrication process to make the micro-bar or a sophisticated program to control the stirring movements.

Several groups have studied the kinematics of these magnetically-actuated droplets. Most of them employed non-reactive droplets to avoid the chaotic chemical reactions from interfering with their observations. One group demonstrated the mixing, merging and splitting of droplets on top of the silicon nanowire surface [13,14,15]. The mobile droplet was inserted with carbonyl iron particles. They documented the effect of varying the viscosity and volume of droplets, and iron particle concentration. Another group [16,17,18] investigated the kinematics of the ferrofluid droplets on top of a Teflon-coated glass plate. In the earlier works, these droplets were driven by magnetic coils [16,17]. They studied the influence of the volume of droplet, its viscosity and the coil’s electric current to the speed and deformation of the moving droplet. In their follow up works, the ferrofluid droplets [18] and ferrofluid marbles [19] were actuated by a permanent magnet instead of coils. In the most recent article on this study from another group, Saroj et al. [20] used four copper coils to mix two fluids on top of a glass substrate. Prior to the mixing, a 0.5 μL rhodamine dye was merged with a 1 μL deionised (DI) water droplet using a micro-syringe. The coils were positioned diametrically opposite of each other, with the merged droplet at the centre. A timing sequence fed the pulsed current to all coils to stir the magnetic beads within the droplet. They were able to demonstrate the relationship between the electric current and frequency of the magnets to the reduced mixing time.

The new kinematics knowledge on the non-reactive droplets gathered in the preceding paragraph should be used to study the reactive droplets, i.e., small biological samples. The main difference is the mixing and merging of these samples will produce chemical reactions. Several groups already reported on the hydrodynamics, kinematics and chemical reactions of the reactive micro-droplets. Tsuji and Muller [21] studied the early stage of the chemical reaction when a droplet of bromothymol blue (16 μL) was laterally impinged on the surface of 0.1 M sodium hydroxide (NaOH) solution. The merging process was recorded using a high speed camera. They observed “finger formation” on the surface of the solution in the first 1.5 s, as shown in Figure 3a of the article. The structure suggested some type of hydrodynamics instabilities of the chemical front on the surface of the droplet. It could be caused by a variety of factors. In that particular work, they credited it to the released heat from the exothermic reaction. Another group i.e., Yeh et al. [22] studied the kinematics of the mixing and merging of phenolphthalein and 0.1 M NaOH micro-droplets. The passive mixing was implemented using different wettability gradient on top of a glass surface. They also mixed two non-reactive droplets i.e., Tartrazine (yellow die) and indigo carmine (blue dye) as the control. The mixing time of the non-reactive was more than 100 times slower than the reactive droplets. For the mixing of the reactive droplets, they recorded the evolution of the chemical reaction that was highly dependent on the “neck curvature”. The definition for this parameter is illustrated in Figure 2b of their article. They concluded that the growth rate of the “finger formation” is inversely proportional to the radius of the “neck curvature”. There is also another group i.e., Torres et al. that reported a complete enthalpy array system [6,7,10,23]. They developed perhaps the most advanced prototype in terms of maturity, scalability and versatility [1]. That system was able to measure the binding and enzymatic reactions of a variety of compounds with high level of sensitivity. We highlighted their major contributions and impacts in our review of the basic calorimeter system [1], and will not repeat the detailed descriptions here. It is worth mentioning that their system was developed in two stages. In the first prototype, an electrostatically-actuated mechanism was employed [6,7]. In the later prototype [10], the mixing time was enhanced by employing the magnetic micro-stirring mechanism. In both systems, a polymer cap was used to cover the measurement chamber to avoid the effect of evaporation. This group has also published a focused review on higher-throughput calorimeters [23].

There is a practical issue in terms of handling small biological samples. In magnetically-actuated microfluidic systems [1], the mobile droplet contains magnetic particles. After the merging is completed and the data is collected, the separation of these biological particles from the magnetic particles is not a straightforward process. This is especially challenging when the droplet is contained in a closed chamber system. That separation is sometimes necessary when the mixed droplet that contains the biological sample needs to be transferred to the next equipment, for example a mass spectrometer for another analysis. On the open chamber system, a micro-pipette could be used to cautiously transfer the liquid from the mixed droplet, since the magnetic particles are heavily concentrated at the bottom of the droplet. In addition, Torres et al. [23] compared the performance of prototyped calorimeters and concluded that the open-chamber configuration had better sensitivity i.e., lower thermal fluctuation. 

The advantages as described in the preceding paragraph open opportunities for further exploration of magnetically-actuated open-surface systems that can measure exothermic reactions. For example, we would like to understand the kinematics of the merging and mixing of highly reactive droplets. In order to do this, a highly exothermic reaction involving ions with large diffusion coefficients is preferred. The works of Almarcha et al. [24] and Zats et al. [25] are referred for this purpose. In both, they studied the hydrodynamics instabilities of acid-base reaction, each having 1.0 M concentration. Their hydrochloric acid (HCl) solution was stacked on the top of NaOH solution in a Hele-Shaw cell. We will use the same concentrated solutions in our studies, but in the form of micro-droplets. They will be horizontally mixed and merged by magnetic actuation. Furthermore, we employ the established apparatus and data acquisition system that was used for our kinematics and dynamics studies of the magnetically-actuated liquid marbles [26,27].

This paper reports the preliminary investigation of the magnetically-actuated mixing and merging of two highly reactive acid-base droplets on top of an open hydrophobic surface. We study the influence of the droplet volume, permanent magnet speed and iron oxide concentration on the mixing time, peak temperature and cooling time. The rest of the paper is divided as follows: Section 2 discusses the experimental setup, data acquisition and materials. Section 3 tabulates the measured data and their trends in term of graphical representations and textual discussions. Section 4 concludes the paper by discussing the limitations of this study and proposes future works.

## 2. Materials and Method

### 2.1. Experimental Setup

**Figure 1:**
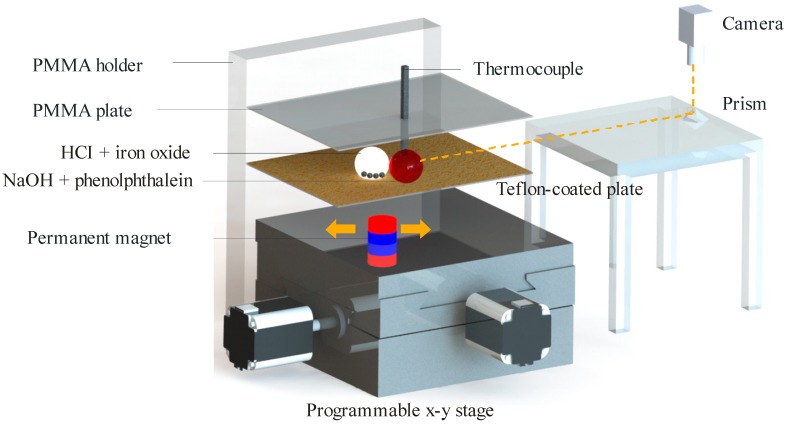
Experimental setup.

Figure 1 illustrates the setup. The major components were a polymethyl methacrylate (PMMA) holder, two PMMA plates, a thermocouple, a permanent magnet and a programmable x-y stage. A vernier caliper was used to measure the mesoscale dimension. All PMMA blocks were designed using CorelDRAW (Corel Corp, Ottawa, ON, Canada) and cut by a laser engraver (Rayjet 300, Trotec Laser, Wels, Austria). The PMMA holder was 4.5 mm thick and had two slits to hold two 1.5 mm thick PMMA plates. The upper plate had a hole (0.5 mm in diameter) to hold a thermocouple (Type K, Pico Technology Limited, St Neots, UK). The tip of the thermocouple was welded in the shape of a sphere (0.2 mm in diameter). The droplets were placed on top of the lower plate. This plate was covered with 1200 grit sandpaper. Teflon powders (Sigma Aldrich, 1 µm particle size, Saint Louis, MO, USA) were coated on the sandpaper to create a hydrophobic surface. The thermocouple was vertically positioned on the top plate, so that its tip touched the sessile droplet on the lower plate.

A cylindrical neodymium magnet (10 mm in diameter, 5 mm in height) was fixed on top of the programmable x-y stage. There was a vertical gap of 2 mm between the magnet and the lower plate. A handheld gaussmeter (GM07, Hirst Magnetic Instruments Ltd., Falmouth, UK) was used to measure the magnetic flux density. Its value on the surface immediately above the magnet was 235 mT. The x-y stage (T-LS28M, Zaber Technologies, Vancouver, BC, Canada) was a motorized linear actuator. This actuator had a travel range of 28 mm, and a minimum and maximum speed of 0.930 and 6.5 mm/s, respectively. The linear motion was controlled by the Zaber console software. The x-y stage was connected to the PC through the RS-232 port. Codes were written to program the range and speed.

### 2.2. Image and Temperature Data Acquisition

The video recording of the droplets was performed by a camera (Edmund Optics Inc., Singapore). It used an adjustable lens (AF Micro Nikkor 60 mm f/2.8D, Nikon, Tokyo, Japan). A horizontal shot was needed to capture the merging and mixing of the droplets. A direct capture using the camera proved to be difficult. This was due to the refraction of light from the PMMA holder. In order to mitigate this problem, a 45° prism was employed, as shown in Figure 1. The yellow dotted line represents the “line of sight” between the camera and the droplets through the prism. The camera lens was set in the “face down” position by attaching it to a PMMA slab (not shown in Figure 1). This slab was in turn clamped to the lab jack. The camera was connected to the PC through a USB port. The camera software (uEye cockpit, IDS, Ettlingen, Germany) set the video recording at 39.2 frames per second and at 82 MHz pixel clock. The image processing of the captured movie is explained in Section 2.4. Three sampled movies are provided as supplementary files S1, S2 and S3.

For the temperature recording, the data logger (Picolog USB TC-08, Pico Technology Limited., St Neots, UK) documented the temperature readings throughout the duration of the experiment. Temperature data were sampled every 1 ms. The video recording and the temperature data acquisition ran simultaneously.

### 2.3. Experimental Details

The micro-droplets were dispensed using a micro-pipette (Genex-Beta, London, UK). That pipette can dispense volumes ranging from 0.5 to 10 μL in a spherical shape. We prepared a 1.0 M solution of hydrochloric acid (HCl 32%, MW = 36.46 g/mol, AjaxFineChem, Thermo Fisher Scientific, Scoresby, VIC, Australia) and a 1.0 M solution of sodium hydroxide (NaOH pellet, MW = 40 g/mol, Chem Supply, Gillman, Australia). The mobile droplet contained the HCl solution and iron oxide particles. The sessile droplet contained NaOH and phenolphthalein (solution 1% in ethanol, Scharlau, Barcelona, Spain) solution. This pH indicator was used to visualize the mixing of the acid-base droplets. We also used the DI water to create the non-reactive droplets as control. A yellow food dye (Queen Fine Food, Alderley, QLD, Australia) was injected into the sessile droplet to visualize the mixing process. In this work, all droplets were dispensed in three volumes, namely 1, 5 and 10 µL.

The mobile droplet contained iron oxide particles (Sigma-Aldrich, Iron II/III oxide, Fe_3_O_4_, MW = 231.53 g/mol). These magnetite particles are spherical with a mean diameter of less than 5 μm. They have an iron content of between 68.0 to 76.7 wt %. They were weighed using a high precision analytical balance (Radwag, Radom, Poland) with readability down to 0.1 mg. For example, 2 mL of HCl fluid was mixed with 20 mg of iron oxide particles to make the 0.010 g/mL concentration. In this experiment, the concentration of the iron oxide particles was fixed to be 0.010, 0.020 and 0.040 g/mL.

Since the Fe_3_O_4_ particles were mixed with HCl, there was a possibility for reaction (1) to occur:

Fe_3_O_4_ + 8HCl → FeCl_2_ + 2FeCl_3_ + 4 H_2_O (1)

This is an exothermic reaction and produces a heat of neutralisation. As a consequence, it may interfere with the measurement of the heat of neutralisation between HCl and NaOH. Several books mention that Fe_3_O_4_ particles dissolve slowly in HCl [28,29,30]. Their inertness is the reason for these particles to be used as a magnetic carrier for catalyst or enzymes [31]. We needed to verify this assumption. Therefore, we mixed the iron oxide particles into the 1.0 M HCl solution and measured the dissolved rate by monitoring the temperature of the solution using the thermocouple.

**Figure 2:**
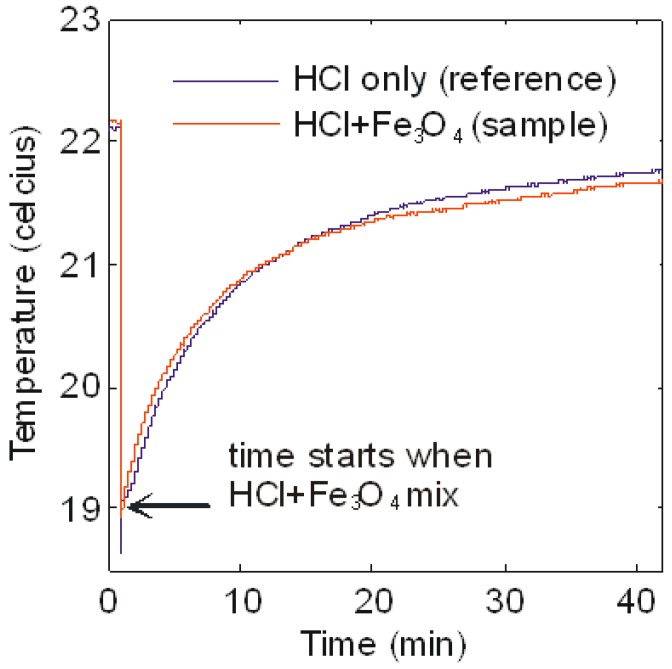
Measurement of the temperature of HCl (blue line) and HCl + Fe_3_O_4_ (red line) over 40 min. We use the highest concentration of the iron oxide particles i.e., 0.040 g/mL.

Figure 2 shows the plot of temperature versus time. The blue and the red lines are the temperature reading of the HCl solution (control) and HCl + Fe_3_O_4_ solution, respectively. Both lines coincide throughout the 40 min duration, indicating indifferent thermal reaction. There is a slow increment of the temperature from 19 to 21° Celsius in this space, reflecting a consistent ambient temperature. Based on this experiment, we could assume that reaction (1) does not influence the measured peak temperature of the exothermic reaction between HCl and NaOH. The slow reaction also means that Fe ions dissolved long after the data for the acid-base reactions have been collected. In other words, the concentration of the iron oxide particles remains constant throughout the primary experiments. 

As shown in Figure 1, the Teflon-coated plate served as the planar surface for the merging and mixing of the droplets. We scratched off the Teflon powder to leave a small hydrophilic area beneath the thermocouple. A pipette was used to dispense the sessile droplet on top of that area. This droplet appeared pink due to the high amount of phenolphthalein ions. Then, a pipette was used to dispense the mobile droplet on the other side of the plate. Since this droplet contained the magnetic particles, a magnet below the surface controlled its motion. The programmable x-y stage moved the magnet, which in turn dragged the mobile droplet towards the sessile droplet in linear motion. In this experiment, four mixing speeds were chosen, namely 1.86, 2.79, 3.72 and 4.65 mm/s. 

**Figure 3:**
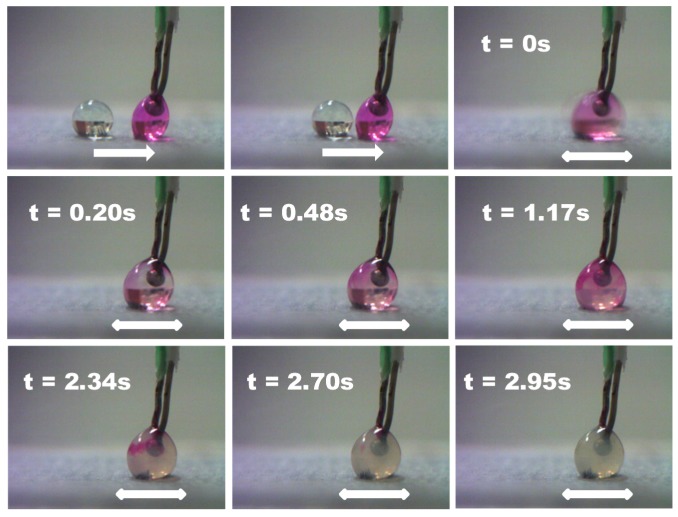
The sequence of frames to illustrate the merging and mixing of the HCl and NaOH droplets.

Mixing started (t = 0 s) when the droplets merged. At this point, the magnet was programmed to move back and forth to speed up the mixing rate. This mixing was deemed to be completed when the mixed droplet was transparent in colour. The exact mechanism to determine the mixing time will be explained in the next section. Throughout the whole experiment, the thermocouple concurrently recorded the temperature of the droplet. Its sphere-shape tip was inside the sessile droplet. Figure 3 illustrates one example of the merging and mixing of two droplets. The white arrow indicates the movements of the magnet. In this figure, the speed of the magnet is 2.79 mm/s and the concentration of the iron oxide particles is 0.010 g/mL. The total mixing time is determined to be 2.95 s.

### 2.4. Determining the Mixing Time

The mixing time was determined as follows. First, we used image analysis software (ImageJ, National Institute of Health, Bethesda, MD, USA) to extract the individual frames from the movie file. Each 8-bit coloured frame with a specific time-stamp was then converted into a grayscale image. Then, we manually cropped smaller image, which focused on the mixing process within the mixed droplet. The area that was dominated by the iron oxide particles was excluded, since their colours remained blackish throughout the mixing process. This cropped image was split into N number of pixels. For each pixel, the software determined its intensity (*c_K_*), ranging from 0 (pure white) to 255 (pure black). The data was transferred to the custom program (MATLAB, Mathworks, Natick, MA, USA). It calculated the average pixel intensity ($\bar{c}$) for that particular frame based on the values of N and *c_K_*. Then, the mixing coefficient (η) was determined from Equation (2):

(2) $$\eta =\left[1-\sqrt{\frac{1}{N}\sum {\left(\frac{c_{k}-\bar{c}}{\bar{c}}\right)}^{2}}\right]$$

The value of η was determined for each frame. Its value would be between 0 and 1, depending on the rate of mixing. Once η reached 1, the first frame that carried that value marked the end of the mixing process. Correspondingly, the time stamp for that frame was the total mixing time. 

## 3. Results and Discussion

We performed a set of experiments to find the following parameters: mixing time, peak temperature and cooling time. They were plotted as a function of the volume of droplets, speed of the magnet and concentration of the iron oxide particles. To ensure consistency in the chemical reaction, we used the identical volume and concentration for the HCl and NaOH droplet. Each plotted data point was the average reading of four repeated experiments. The error bar represents the fluctuation between these individualised data. Since we had proven that the exothermic reaction of Fe_3_O_4_ dissolution in HCl did not influence the acid-base neutralization as shown in Figure 2, we assumed that there was no dissolution of Fe ions with the experiment time frame. Thus, the concentration of iron oxide particles remained constant throughout the whole experiment. In the first experiment, we will compare the mixing time of the reactive and non-reactive droplet, as described next.

### 3.1. Mixing Time of the Reactive and Non-Reactive Droplets

**Figure 4:**
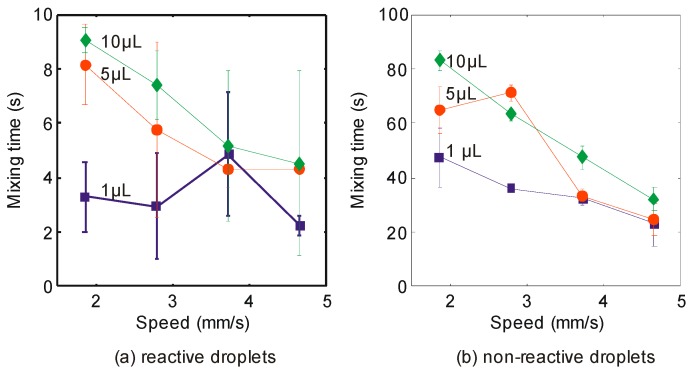
The y-axis represents the mixing time of (**a**) reactive droplets and (**b**) non-reactive droplets. The x-axis represents the incremental speed of the magnet (1.86, 2.79, 3.72 and 4.65 mm/s). The blue, red and green plots represent the volume (1, 5 and 10 μL) of the droplets, respectively. The concentration of the iron oxide particles is fixed at 0.010 g/mL.

The mixing time is important to determine the speed of mixing (sometimes referred to as coalescence in literature) between two droplets. In this experiment, we study the influence of the speed of the magnet and the volume of the droplets to this parameter. Figure 4 shows the mixing time of the reactive and non-reactive droplets. The first observation is as follows. The mixing time is reduced with the increasing speed of the magnet and the decreasing volume of droplets. Both trends are consistent in either the reactive or non-reactive mixing. Many studies reported these trends for the non-reactive droplets. There are two outliers in our experiment. First, the reactive droplets with the volume of 1 µL at the speed of 3.72 mm/s. Second, the non-reactive droplets with the volume of 5 µL at the speed of 2.79 mm/s. While the chemical reaction could be the possible reason for the former, it could not be the reason for the latter. The second observation is more profound. The mixing time of the reactive droplets is 7 to 12 times lower than the non-reactive droplets. Similar trend had been reported by Yeh et al. [22]. Their mixing time of the non-reactive droplet was 100 times lower than their reactive droplets. The reasons were because their system used a passive mixing and a lower concentration of 0.1 M of NaOH.

### 3.2. Measuring Peak Temperature and Determining Cooling Time

Next, we focus on the reactive droplets. We measure the peak temperatures and extract their cooling time. The peak temperature (*T*_0_) could be used to characterize the energy that is released during the exothermic reaction of the acid-base neutralization. A thermocouple was used to directly measure this parameter. The peak temperature is determined after the mixing has completed i.e., η = 1. It should be noted that in all the graphs, the absolute value of the peak temperature is not given. Instead, the relative (sometimes referred to as normalized in the literature) value i.e., the difference between the peak temperature and the ambience temperature during the experiment is provided. 

**Figure 5:**
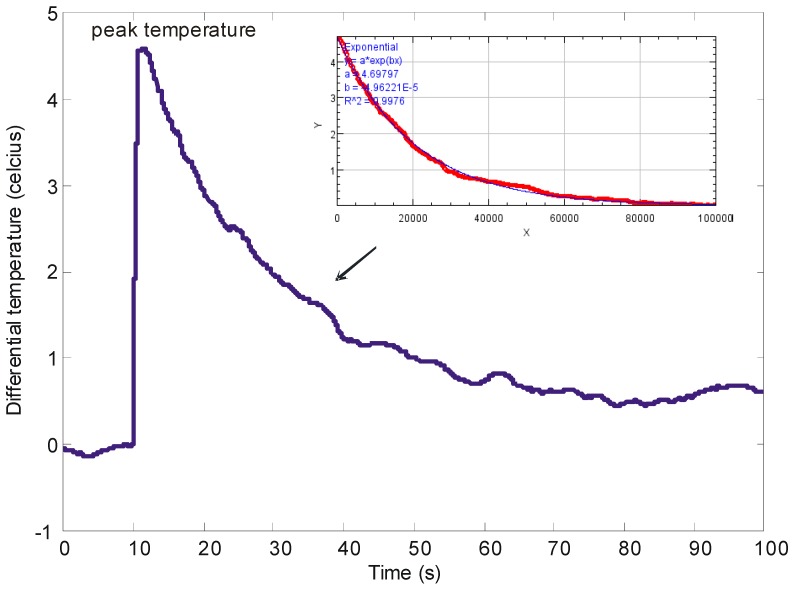
The y-axis represents the difference between the peak temperature and ambience temperature, referred to as relative temperature in this paper. In this particular example, the exothermic reaction produces almost a 5 °C rise in temperature. Once the exothermic reaction is completed, the droplet cools down exponentially to reach equilibrium. The inset figure shows the curve fitted graph to find the cooling time.

The cooling time could be used to determine the minimum waiting period before the mixed droplets could be transferred out for other characterizations. Figure 5 shows one example of the exponential-decay from the peak temperature of the exothermic reaction to the ambience temperature. It is obvious that the tail of the graph fluctuates over time. Therefore, we could not determine the cooling time from it. Instead, we estimate the value of this parameter by using a curve fitting toolbox (cftool) from MATLAB (Mathworks). This example of the curve-fitted graph is shown in the inset of Figure 5. Based on the exponential-decay equation, the tool automatically finds the values of *a* and *b*. The best result is found by maintaining the R-squared value as close to 1. Then, the values of *a* and *b* are mapped to the larger plot using Equation (3):

(3) $$T\left(t\right)=T_{0}e^{-\frac{t}{\tau }}$$

where y = *T(t)* is the temperature at time *t*, *a* = $T_{0}$ is the peak temperature and *b* = 1/*τ* is the inverse of the cooling time. It should be noted that the value of *a* is made as close as possible to *T*_0_. 

### 3.3. The Influence of Speed of Magnet and Concentrations of Iron Oxide Particles on the Peak Temperature and Cooling Time

**Figure 6:**
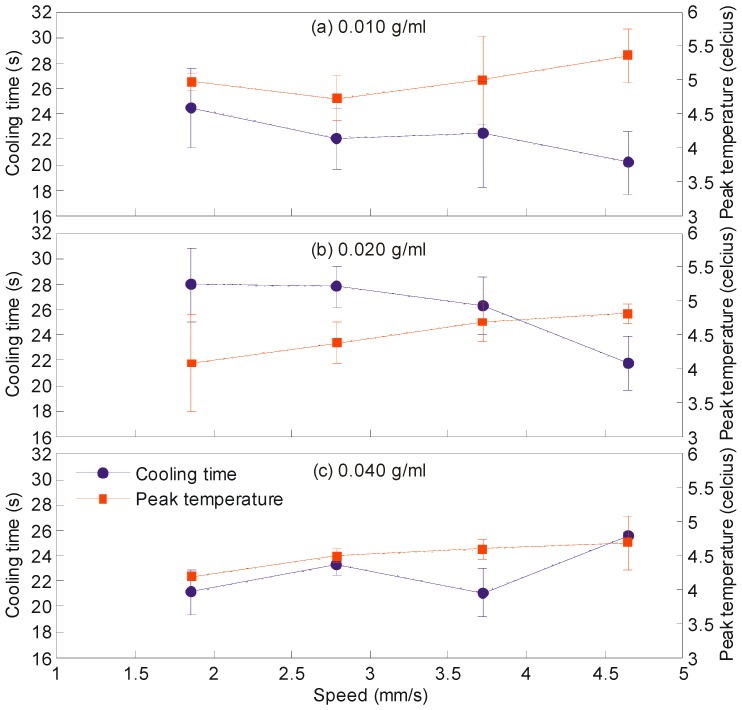
These plots show the trend of the cooling time (blue line) and relative peak temperature (orange line) with reference to the speed of the magnet. The four horizontal points represent the speed of 1.86, 2.79, 3.72 and 4.65 mm/s. Panel (**a**–**c**) represent the trend at three different concentrations of iron oxide particles. All the droplets have the volume of 5 µL.

As presented in Section 3.1, the speed of magnet influenced the mixing rate. It was deduced that the higher the speed, the faster the mixing was completed. Correspondingly, we would like to study its influence on the peak temperature and cooling time on the reactive droplets with the fixed volume of 5 µL. In addition, we vary the concentration of the iron oxide particles to see if it influences the heating and cooling effects on the droplets. Figure 6 shows the results. The general trends are as follows. As the speed of the magnet increases, the peak temperature increases and the cooling time decreases. The trend on the peak temperature is explained as follows. The acceleration of the magnet and the increased momentum from the additional mass of the magnetic particles contribute to an additional kinetic energy to the mobile droplet. This energy is translated into heat during the merging (sometimes referred to as collision in the literature) with the sessile droplet. The trend on the cooling time is explained as follows. As the magnet accelerates during the merging process, the increased circulation of the liquid cools down the droplet faster. It should be highlighted that this trend is not evident from panel (c) of Figure 6. The cooling time fluctuates for the droplet that has the iron oxide particles with the concentration of 0.040 g/mL. Note that the average cooling time for this particular droplet is the lowest compare to the other two. It indicates the positive influence of the higher magnetic to the heat transfer within that droplet. Further experiments need to be performed to find the reason(s) for this fluctuation. The work of Roy et al. [12] can be referred. They characterized the circular movements of the magnetic particles within the non-reactive droplet during the stirring process. Similar study could be made for the reactive droplet.

### 3.4. The Influence of Volume of Droplets on the Peak Temperature and Cooling Time

**Figure 7:**
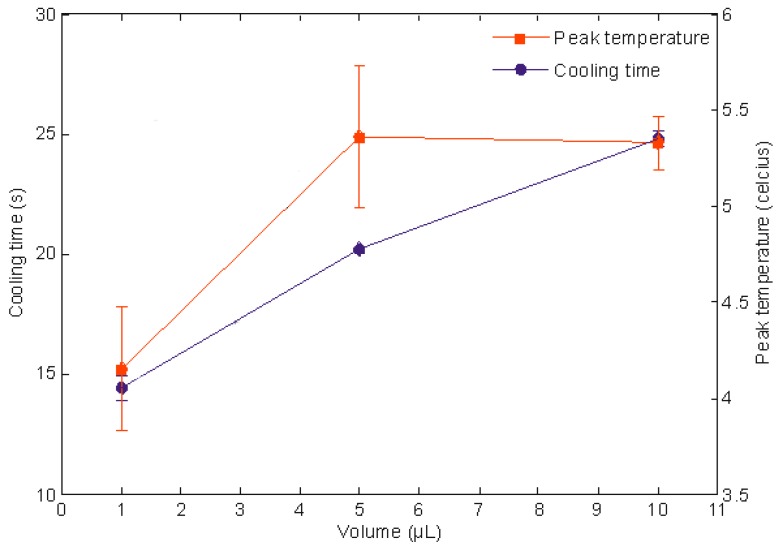
This graph shows the cooling time (blue line) and relative peak temperature (orange line) with reference to the volume of the droplets. The three dots and squares represent the volumes of 1, 5 and 10 µL. In all three droplets, the iron oxide concentration and speed of the magnet are fixed at 0.01 g/mL and 4.65 mm/s, respectively.

In theory, the peak temperature is largely dependent on the speed of the magnet and the concentration of the acid-base droplets. The volume of the droplet should not influence this parameter. On the other hand, the same parameter positively influences the cooling time. That is, the larger cooling time is needed for the droplet with a higher volume. Both hypotheses are tested in this section. Three volumes were prepared i.e., 1, 5 and 10 µL and we used the identical volume for the mobile and sessile droplets. The concentration of the iron oxide particles and the speed of the magnet were kept constant at 0.010 g/mL and 4.65 mm/s, respectively. Figure 7 illustrates the trends. The larger droplets takes longer to cool down, which is in agreement with the theory. The relative peak temperatures for the 5 and 10 µL are similar. However, the value of the peak temperature is much smaller for the 1 µL. One of the possible reasons is because the small droplet cools much faster than the larger droplets due to the high rate of evaporation [32].

## 4. Conclusions

This paper documented the magnetically-actuated merging and mixing of 1.0 M acid-base micro-droplets on top of an open surface. Videos S2 and S3 are provided as supplementary files to illustrate these processes. We observed that even in the presence of the highly diffusive chemical reaction, these droplets followed the widely reported kinematics trends of the non-reactive droplets. The lower volume of the droplet and the higher speed of the magnet led to the shorter mixing time. The acid-base reaction produced heat of neutralisation. Therefore, we studied the influence of the volume of droplet, speed of the magnet and concentration of the iron oxide particles to the peak temperature and the droplet’s cooling time. We found that the peak temperature increased at the higher speed of the magnet and remained consistent for the bigger droplets. In addition, the cooling time decreased at the higher speed of magnet and increased at the higher volume of the droplets. 

In the course of conducting these experiments, we also realized the limitations. In terms of the apparatus, two areas needed to be addressed. First, the use of a large thermocouple provided path for conduction of heat from the micro-droplets. In future, it should be replaced with the on-chip thermometer being embedded on the surface [1] or by using a thermal camera. Second, the effect of evaporation that was reported for the 1 µL droplet in Section 3.4 needed to be addressed. There are two options. The droplets that were used in this experiment could be coated with silicon oil to maintain the humidity. Alternatively, a liquid marble could be used instead. Our study of the marble [26] indicated that it has lower evaporation rate. We refer to Figure 2 of that paper for details. 

In terms of our current results, the characterizations of the chemical reaction of these magnetically actuated acid-base droplets would be useful. As mentioned in Section 1, Yeh et al. [22] passively merged and mixed phenolphthalein and 0.1 M NaOH micro-droplets. They studied the chemical reaction at the interface of the mixed droplet using a high speed camera. Our future works should follow that characterization procedure. The variation of the volume of the acid and base droplets would produce different profiles of the “neck curvature” during the merging of the droplet [22]. Ultimately we would like to record the “finger formation” as reported by several articles that studied the hydrodynamics instabilities of those reactions [21,22,24,25]. In addition, the concentration of the acid-base solutions should be titrated from 1.0 M down to a micro-M. By doing that, we could study the influence of these lower concentrated droplets to the measured peak temperature. It is crucial to determine the fluctuation of this peak temperature with reference to the droplets that have the micro-M to milli-M concentrations for practical applications [6,7,10,23,33].

## Supplementary Materials

The following videos are available online at http://www.mdpi.com/1424-8220/18/6/1767/s1.Video S1: This video shows the merging and mixing of two non-reactive droplets via magnetic actuation. Both contains DI water with a volume of 10 μL. The mobile droplet has an iron oxide concentration of 0.040 g/mL. The magnet moves at a speed of 4.65 mm/s. The yellow food dye is injected to the mobile droplet to visualize the mixing and merging processes. The mixing time is calculated when the mixed droplet has consistent yellow colour. Video S2: This video shows the merging and mixing of two reactive droplets via magnetic actuations. Both has a volume of 1 μL. The mobile droplet contains 1.0 M HCl solution + iron oxide particles, and the sessile droplet contains 1.0 M NaOH + phenolphthalein. The former has transparent color with a blackish iron oxide at its bottom, with a concentration of 0.040 g/mL. The latter appears pinkish initially due to the phenolphthalein. This pH indicator was used to visualize the mixing of the acid-base droplets. The magnet moves at a speed of 1.86 mm/s. The mixing time is calculated when the mixed droplet has transparent colour. Video S3: This video illustrates similar processes as in S2, except that the volume of both droplets is 5 μL and the speed of the magnet is 2.79 mm/s. The iron oxide concentration remains the same i.e., 0.040 g/mL. This video also shows the mechanism for the measurement of the exothermic reaction between the HCl and NaOH droplets. A thermocouple is inserted inside the sessile droplet before the mixing begins. It continues to record the temperature measurement every 1 ms until the mixing and merging processes have been completed. Figure 3 shows the frame-by-frame illustration of similar processes, but with the iron oxide concentration of 0.010 g/mL.

## References

[1] Khaw M.K., Mohd-Yasin F., Nguyen N.T. (2016). Microcalorimeter: Design considerations, materials and examples. Microelectron. Eng..

[2] Demello A.J. (2006). Control and detection of chemical reactions in microfluidic systems. Nature.

[3] Nguyen N.T., Wu Z. (2005). Micro-mixers—A review. J. Micromechan. Microeng..

[4] Nguyen N.T., Wereley S. (2006). Fundamentals and Applications of Microfluidics.

[5] Suh Y.K., Kang S. (2010). A review on mixing in microfluidics. Micromachines.

[6] Torres F.E., Kuhn P., de Bruyker D., Bell A.G., Wolkin M.V., Peeters E., Williamson J.R., Anderson G.B., Schmitz G.P., Recht M.I. (2004). Enthalpy Array. Proc. Natl. Acad. Sci. USA.

[7] Recht M.I., de Bruyker D., Bell A.G., Wolkin M.V., Peeters E., Anderson G.B., Kolatkar A.R., Bern M.W., Kuhn P., Bruce R.H. (2008). Enthalpy array analysis of enzymatic and binding reactions. Anal. Biochem..

[8] Long Z., Shetty A.M., Solomon M.J., Larson R.G. (2009). Fundamentals of magnet-actuated droplet manipulation on an open hydrophobic surface. Lab Chip.

[9] Nguyen N.T., Zhu G., Chua Y.C., Phan V.N., Tan S.H. (2010). Magnetowetting and sliding motion of a sessile ferrofluid droplet in the presence of a permanent magnet. Langmuir.

[10] de Bruyker D., Recht M.I., Bhagat A.A.S., Torres F.E., Bell A.G., Bruce R.H. (2011). Rapid mixing of sub-microlitre drops by magnetic micro-stirring. Lab Chip.

[11] Lu L.H., Ryu K.S., Liu C. (2002). A magnetic microstirrer and array for microfluidic mixing. J. Microelectromechan. Syst..

[12] Roy T., Sinha A., Chakraborty S., Ganguly R., Puri I.K. (2009). Magnetic microsphere-based mixers for microdroplets. Phys. Fluids.

[13] Egatz-Gómez A., Melle S., García A.A., Lindsay S.A., Márquez M., Domínguez-García P., Rubio M.A., Picraux S.T., Taraci J.L., Clement T. (2006). Discrete magnetic microfluidics. Appl. Phys. Lett..

[14] Garcia A.A., Egatz-Gómez A., Lindsay S.A., Dominguez-Garcia P., Melle S., Marquez M., Rubio M.A., Picraux S.T., Yang D., Aella P. (2007). Magnetic movement of biological fluid droplets. J. Magn. Magn. Mater..

[15] Schneider J., Egatz-Gómez A., Melle S., Lindsay S., Dominguez-Garcia P., Rubio M.A., Marquez M., García A.A. (2008). Motion of viscous drops on superhydrophobic surfaces due to magnetic gradients. Colloids Surf. A Physicochem. Eng. Aspects.

[16] Nguyen N.T., Ng K.M., Huang X. (2006). Manipulation of ferrofluid droplets using planar coils. Appl. Phys. Lett..

[17] Nguyen N.T., Beyzavi A., Ng K.M., Huang X. (2007). Kinematics and deformation of ferrofluid droplets under magnetic actuation. Microfluid. Nanofluid..

[18] Koh W.H., Lok K.S., Nguyen N.T. (2013). A digital micro magnetofluidic platform for lab-on-a-chip applications. J. Fluids Eng..

[19] Nguyen N.T. (2013). Deformation of ferrofluid marbles in the presence of a permanent magnet. Langmuir.

[20] Saroj S.K., Asfer M., Sunderka A., Panigrahi P.K. (2016). Two-fluid mixing inside a sessile micro droplet using magnetic beads actuation. Sens. Actuators A Phys..

[21] Tsuji K., Müller S.C. (2012). Chemical reaction evolving on a droplet. J. Phys. Chem. Lett..

[22] Yeh S.I., Sheen H.J., Yang J.T. (2015). Chemical reaction and mixing inside a coalesced droplet after a head-on collision. Microfluid. Nanofluid..

[23] Torres F.E., Recht M.I., Coyle J.E., Bruce R.H., Williams G. (2010). Higher throughput calorimetry: Opportunities, approaches and challenges. Curr. Opin. Struct. Biol..

[24] Almarcha C., Trevelyan P.M., Grosfils P., de Wit A. (2010). Chemically driven hydrodynamic instabilities. Phys. Rev. Lett..

[25] Zalts A., el Hasi C., Rubio D., Urena A., D’Onofrio A. (2008). Pattern formation driven by an acid-base neutralization reaction in aqueous media in a gravitational field. Phys. Rev. E.

[26] Khaw M.K., Ooi C.H., Mohd-Yasin F., Vadivelu R., John J.S., Nguyen N.T. (2016). Digital microfluidics with a magnetically actuated floating liquid marble. Lab Chip.

[27] Khaw M.K., Ooi C.H., Mohd-Yasin F., Nguyen A.V., Evans G.M., Nguyen N.T. (2017). Dynamic behavior of a magnetically actuated floating liquid marble. Microfluid. Nanofluid..

[28] Sigmund W., El-Shall H., Shah D.O., Moudgil B.M. (2008). Particulate Systems in Nano- and Biotechnologies.

[29] Talbot D.E., Talbot J.D. (2010). Corrosion Science and Technology.

[30] Dulski T.R. (1996). A Manual for the Chemical Analysis of Metals.

[31] Cheng R., Li G.-Q., Cheng C., Shi L., Zheng X., Ma Z. (2015). Catalytic oxidation of 4-chlorophenol with magnetic Fe_3_O_4_ nanoparticles: Mechanisms and particle transformation. RSC Adv..

[32] Kovalchuck N.M., Trybala A., Starov V.M. (2014). Evaporation of sessile droplets. Curr. Opin. Colloid Interface Sci..

[33] Lehmann U., de Courten D., Vandevyver C., Parashar V.K., Gijs M. (2009). On chip antibody handling and colorimetric detection in a magnetic droplet manipulation system. Microelectron. Eng..

